# Identification of *in vivo* induced antigens of the malacosporean parasite *Tetracapsuloides bryosalmonae* (Cnidaria) using *in vivo* induced antigen technology

**DOI:** 10.3389/fcimb.2022.1032347

**Published:** 2022-10-26

**Authors:** Gokhlesh Kumar, Arun Sudhagar, Saloni Shivam, Frank Nilsen, Jerri L. Bartholomew, Mansour El-Matbouli

**Affiliations:** ^1^ Clinical Division of Fish Medicine, University of Veterinary Medicine Vienna, Vienna, Austria; ^2^ Peninsular and Marine Fish Genetic Resources Centre, Indian Council of Agricultural Research (ICAR) – National Bureau of Fish Genetic Resources, Kochi, India; ^3^ Karwar Regional Station of Indian Council of Agricultural Research (ICAR)-Central Marine Fisheries Research Institute, Karwar, India; ^4^ Sea Lice Research Centre, Department of Biological Sciences, University of Bergen, Bergen, Norway; ^5^ Department of Microbiology, Oregon State University, Corvallis, OR, United States; ^6^ School of Biotechnology, Badr University in Cairo, Badr City, Cairo, Egypt

**Keywords:** myxozoa, proliferative kidney disease (PKD), *Tetracapsuloides bryosalmonae*, antigen, *in vivo* induced antigens (IVIAT), host-parasite interaction, salmonids

## Abstract

*Tetracapsuloides bryosalmonae* is a malacosporean endoparasite that causes proliferative kidney disease (PKD) in wild and farmed salmonids in Europe and North America. The life cycle of *T. bryosalmonae* completes between invertebrate bryozoan and vertebrate fish hosts. Inside the fish, virulence factors of *T. bryosalmonae* are induced during infection or interactions with host cells. *T. bryosalmonae* genes expressed *in vivo* are likely to be important in fish pathogenesis. Herein, we identify *in vivo* induced antigens of *T. bryosalmonae* during infection in brown trout (*Salmo trutta*) using *in vivo* induced antigen technology (IVIAT). Brown trout were exposed to the spores of *T. bryosalmonae* and were sampled at different time points. The pooled sera were first pre-adsorbed with antigens to remove false positive results. Subsequently, adsorbed sera were used to screen a *T. bryosalmonae* cDNA phage expression library. Immunoscreening analysis revealed 136 immunogenic *T. bryosalmonae* proteins induced in brown trout during parasite development. They are involved in signal transduction, transport, metabolism, ion-protein binding, protein folding, and also include hypothetical proteins, of so far unknown functions. The identified *in vivo* induced antigens will be useful in the understanding of *T. bryosalmonae* pathogenesis during infection in susceptible hosts. Some of the antigens found may have significant implications for the discovery of candidate molecules for the development of potential therapies and preventive measures against *T. bryosalmonae* in salmonids.

## Introduction

*Tetracapsuloides bryosalmonae* is an endoparasitic myxozoan that completes its life cycle between an invertebrate (bryozoan) and a vertebrate (salmonid fish) host. The parasite causes proliferative kidney disease (PKD) in various species of salmonids (Anderson et al., 1999; Feist et al., 2001) and is reported in Europe and North America, leading to severe losses in trout farms (Hedrick et al., 1993). While the economic impact of the disease makes it an important factor for aquaculture (Clifton-Hadley et al., 1986), PKD is also suspected to be a factor contributing to the decline of wild brown trout and salmonid populations in Europe (Wahli et al., 2002; Sterud et al., 2007; Okamura et al., 2011; Skovgaard and Buchmann, 2012; Dash and Vasemägi, 2014; Vasemägi et al., 2017; Waldner et al., 2020). In Austria, a 92% overall prevalence of *T. bryosalmonae* was detected among wild brown trout sampled from the river Wulka, indicating a possible reason for their decline in this river. The findings suggest that wild brown trout might soon be extinct in the river Wulka of Austria (Waldner et al., 2020). In North America, *T. bryosalmonae* killed thousands of mountain whitefish (*Prosopium williamsoni*) in Montana’s Yellowstone River in August 2016, with a 90% mortality rate, which suggested *T. bryosalmonae* is endemic in Montana (Hutchins et al., 2021) and can cause impacts on native salmonid populations.

The ability of a pathogen to cause disease depends primarily on its ability to sense and adapt to a variety of host environmental signals. A parasite, while invading its host, senses the *in vivo* environment by inducing or repressing the expression of specific genes. A repetitive cycle of cell invasion and replication stimulates the expression of these parasite genes, resulting in disease. Thus, parasite genes expressed *in vivo* are likely to be important virulence mechanisms and potential therapeutic targets. For example, serine and cysteine protease genes of the myxozoan *Myxobolus cerebralis* induced in fish during parasite development are involved in host tissue invasion, virulence, and the initiation of sporogenesis (Kelley et al., 2003; Kelley et al., 2004; Dörfler and El-Matbouli, 2007). In *Ceratonova shasta*, genes associated with parasite adhesion and migration are found to be responsible for the enhancement of virulence in the parasite (Alama-Bermejo et al., 2019). In addition, single nucleotide polymorphism in the motility and protease genes of *C. shasta* affects the outcome of virulence of the parasite in the salmonid host (Alama-Bermejo et al., 2020). Microneme protein MIC11, dense granule protein 5, and calmodulin of *Toxoplasma gondii* are involved in cell division and parasitophorous vacuole maintenance/parasite survival after cellular invasion and pathogenesis in pigs (Tao et al., 2014). The identification of *T. bryosalmonae* virulence genes would improve our understanding of *T. bryosalmonae* infection and promote the discovery of novel therapeutic targets, as well as provide insights into the infection process of the parasite. For this reason, in this study we aimed to identify *T. bryosalmonae* genes that are induced in brown trout during the course of infection. This was achieved using an *in vivo* induced antigen technology (IVIAT), an immunoscreening technique that identifies parasite antigens expressed during infection of the fish host. This approach used pooled serum from brown trout infected with the *T. bryosalmonae* to identify *in vivo* induced (IVI) genes expressed during infection.

## Materials and methods

### *T. bryosalmonae* collection

Our laboratory maintains the life cycle of *T. bryosalmonae* between fish and bryozoan hosts according to Kumar et al. (2013), where we regularly hatch statoblasts and grow bryozoan colonies, harvest parasite sacs and infect brown trout, and cohabitate specific pathogen free *Fredericella sultana* colonies with infected brown trout. For this study, a large number of *T. bryosalmonae* sacs (n ≈ 20,000) were collected from the laboratory-infected colonies by manual microdissection under a stereomicroscope, then transferred to a Petri plate filled with water. The clean isolated parasite sacs were pipetted into 2 ml Eppendorf tubes and centrifuged at 5000 X g for 5 min. The parasite pellets were stored at -80°C for antigen preparation or directly resuspended in an RLT buffer containing β-mercaptoethanol for RNA extraction. Similarly, *T. bryosalmonae* infected *F. sultana* zooids were collected in RNAlater and stored at -20°C for further molecular studies.

### *T. bryosalmonae* expression library

**Figure 1:**
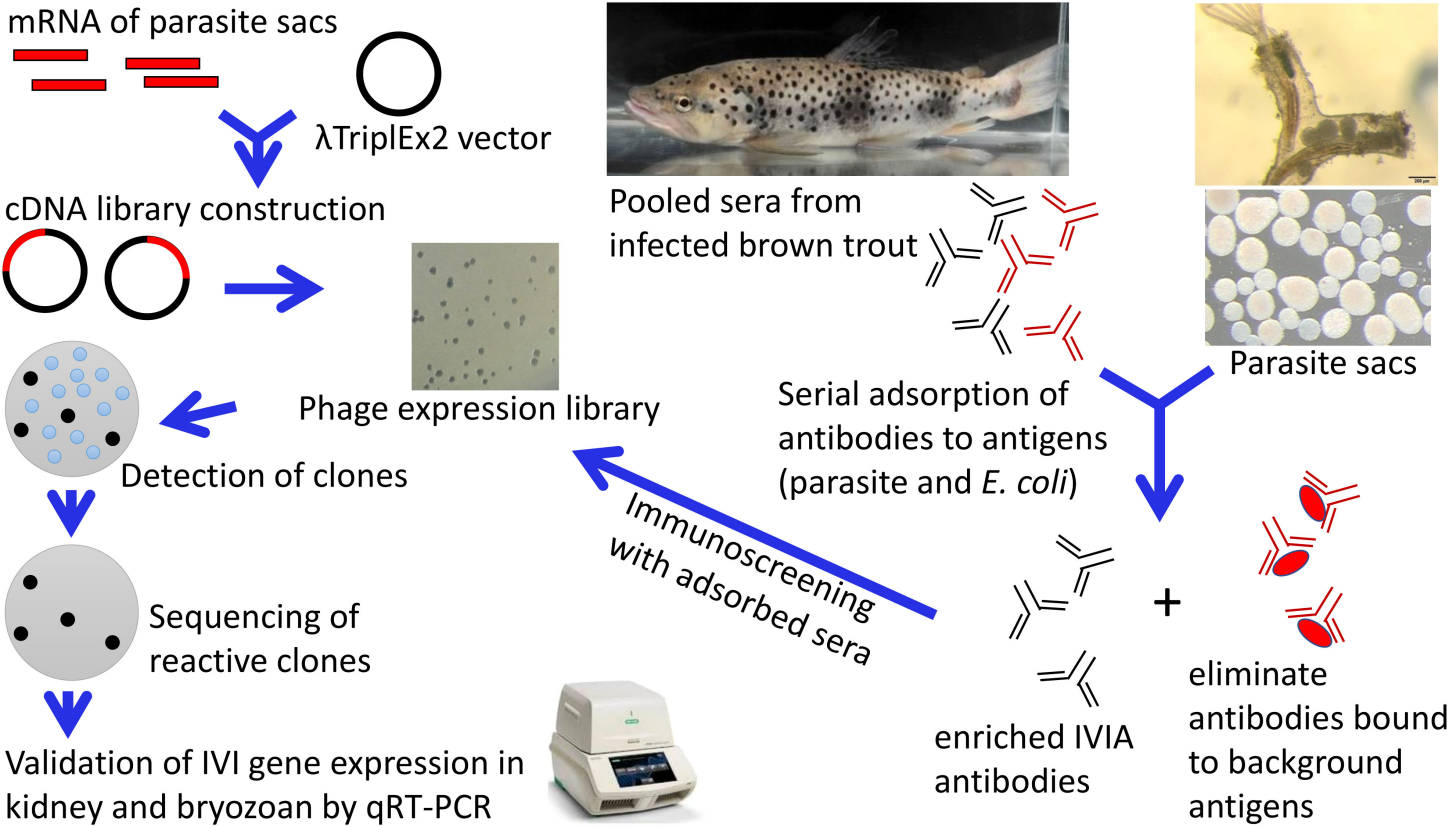
*In vivo*-induced antigen technology applied to *Tetracapsuloides bryosalmonae*. An expression library of *T. bryosalmonae* was constructed into the λTriplEx2 vector. Adsorption of pooled sera were performed to remove antibodies against three forms of parasite sacs and *E. coli* XL1-Blue antigens (native, ultrasonic, and heat-denatured). This process eliminates antibodies in the sera that react to antigens constitutively expressed by *T. bryosalmonae* in the bryozoan body cavity and eliminates background that may arise from the *E. coli*. Immunoscreening of expression cDNA library was performed with adsorbed pooled sera. Positive clones were sequenced, subcloned and validated in infected kidney of brown trout and infected bryozoan by qRT-PCR.

The *in vivo* induced genes of *T. bryosalmonae* in brown trout host were identified using IVIAT and the steps used in this study are presented in a flowchart (**Figure 1**). Total RNA was extracted from the frozen parasite sacs using the RNeasy mini kit (Qiagen, Hilden, Germany). An on-column DNase (Qiagen) digestion step was included according to the manufacturer’s protocol. RNA integrity was measured on the 4200 TapeStation using the RNA Screen Tape assay (Agilent Technologies, USA). Messenger RNA was purified from the extracted RNA (18 µg) sample using the Oligotex mRNA kit (Qiagen) according to the manufacturer’s protocol.

One hundred nanograms of mRNA were synthesized into first-strand cDNA using the SMART cDNA synthesis kit (Clontech Laboratories, USA), then into second strand cDNA by using an LD-PCR protocol. Subsequently, digestion and fractionation steps were performed. The resulting cDNA library was ligated into λTriplEx2 vector (Clontech Laboratories) and then packed in a high-efficiency system using Gigapack III Gold Packaging Extract (Agilent Technologies, USA) according to the manufacturer’s instructions. Resulting library was amplified and titered. To determine the percentage of recombinant clones, blue–white screening was performed on LB/MgSO4 agar plates containing IPTG and X-gal. Thirty clones were randomly picked and tested by PCR analysis using the vector primers (F 5` CTCGGGAAGCGCGCCATTGTGTTGGT 3` and R 5` TAATACGACTCACTATAGGGCG 3`). PCR amplicons were sequenced at the LGC Genomics GmbH (Berlin, Germany) to determine the presence of an insert.

### Sera samples

The sera were previously collected from brown trout infected with *T. bryosalmonae* at 2, 4, 6, 8, 10, 12, and 17 weeks post exposure (wpe) (Sudhagar et al., 2019). Kidney samples were also collected at the same timepoints in RNAlater and stored at -20 °C for molecular studies. Individual serum samples were tested for the presence of *T. bryosalmonae* specific antibodies using indirect ELISA (Shivam et al., 2021). Anti-*T. bryosalmonae* antibody was first detected at 4 wpe and persisted until 17 wpe, thus, these sera were selected for this study. For use in IVIAT, equal volumes of each serum sample were pooled and adsorbed with different antigens as described below. Serum samples from specific pathogen free brown trout that were negative for *T. bryosalmonae* were used to verify the specificity of selected clones. All the serum samples were stored at -80°C until use.

### Preparation of antigens

Three forms of parasite sacs and *E. coli* XL1-Blue antigens were prepared: native, heat-denatured, and non-heat-denatured cell lysates. Parasite sacs and *E. coli* XL1-Blue cells were suspended in Tris-HCl EDTA buffer containing complete protease inhibitor cocktails (Roche, Germany). Both suspensions were divided into three portions. To prepare the non-heat-denatured antigens, parasite sacs and *E. coli* XL1-Blue cells were freeze-thawed six times in liquid nitrogen followed by six rounds of sonication on ice for 10 seconds at 10 Hz. To prepare the heat-denatured antigens, both parasite sacs and *E. coli* XL1-Blue cells were placed at 95°C for 10 minutes.

### Adsorption of sera

Adsorption of pooled sera was performed to remove antibodies against parasite sacs and *E. coli* XL1-Blue. This procedure removes antibodies in the sera that react to antigens constitutively expressed by *T. bryosalmonae* in the bryozoan body cavity and eliminates any background or false positives that may arise from the *E. coli* host strain provided with the SMART cDNA library construction kit. The pooled sera from infected brown trout were adsorbed with native, heat-denatured, and non-heat-denatured cell lysates. Native parasite sacs and *E. coli* XL1-Blue cells were resuspended in pooled sera with the addition of 10 mM EDTA and a complete cocktail of protease inhibitors. The sera-parasite sacs suspension was mixed overnight with gentle rocking (10 rpm) at 4°C. The absorbed sera were recovered by centrifugation at 5,000 x g for 10 minutes at 4°C. Afterwards, heat-denatured and non-heat denatured cell lysates of parasite sacs and *E. coli* XL1-Blue were immobilized on Protran nitrocellulose membranes 0.45 μm (Sigma Aldrich, Germany) overnight at 4°C on a rocking table. Membranes were dried on Whatman papers to remove the excess buffer. Subsequently, membranes were rinsed 5 times in 10 ml of Tris-Buffered Saline (TBS) composed of 10 mM Tris–HCl (pH 7.5) and 150 mM NaCl containing Tween-20 (0.05%). The membranes were blocked by immersion for one hour in 10 ml of 5% immunoblot blocking reagent (Sigma Aldrich) in TBS-T at room temperature on a rocking table. Then membranes were rinsed three times with 10 ml of TBS-T. The pooled sera were incubated on top of the lysate-coated membranes at 4°C overnight with gentle rocking (10 rpm). The same adsorption processes were performed in parallel with pooled sera from non-infected control brown trout. Each adsorption procedure was repeated three times. The final adsorbed sera were centrifuged at 5,000 x g for 10 minutes at 4°C and absorbed sera were recovered by aspiration. A 100 µl aliquot of absorbed sera was collected after the adsorption step to evaluate the efficacy of the process. The remaining adsorbed sera were aliquoted and stored at -80°C. The efficiency of the adsorbed sera from each step were tested by indirect ELISA according to the previously optimized protocol (Shivam et al., 2021).

### Immunoscreening of *T. bryosalmonae* cDNA library

For primary screening, 10 µl of 1:10,000 diluted cDNA library was mixed with 500 µl of overnight culture of *E. coli* XL1-Blue suspended in 7.5 ml of 10mM MgSO_4_ and incubated at 37°C for 15 min. Five hundred microlitre melted (45°C) LB/MgSO4 top agar was added to the phage-bacteria mixture. Subsequently, the mixture was poured immediately onto a pre-warmed (37°C) LB/MgSO4 agar plate (150 mm size) and swirled until the surface of the plate was covered. The plates were incubated at 37°C overnight. The next day, plates were overlaid with Protran nitrocellulose membranes (132 mm size) pre-soaked in IPTG (10 mM), which were left for 5 hours at 37°C to transfer phage particles to the membranes. Afterwards, membranes were rinsed 5 times with TBS-T and blocked with 5% immunoblot blocking reagent for 2 hours at room temperature before being incubated with the pooled optimized adsorbed sera (1:100, diluted in 1% BSA - Dulbecco′s phosphate buffered saline) overnight at 4°C with gentle rocking. Subsequently, membranes were washed with TBS-T five times for 5 min and then incubated with rabbit anti-salmonid polyclonal antibody, Bio-Rad (1:5,000) for 1 hour at 37°C. After washing, peroxidase-conjugated anti-rabbit IgG (whole molecule) antibody, Sigma Aldrich (1:8,000) was applied for 1 hour at 37°C and washed.

The immunoblots were developed using membrane substrate (Sigma Aldrich) according to the user manual. Reactive clones were identified manually by comparing with control and negative control sera. Positive plaques were removed from the gel as a plug, using a cut-off end of a 200 μl pipette tip and then placed into 500 μl of SM buffer containing 3% chloroform. Phages were allowed to diffuse into SM buffer overnight at 4°C. After centrifugation, the supernatants were transferred to new tubes and stored at 4°C. Each positive plaque was screened at least two additional times to confirm that it reacts with the adsorbed sera. *In vivo* excision was performed to convert the recombinant phages to recombinant plasmids according to the user manual. The inserts of *T. bryosalmonae* were determined by PCR amplification using the vector primers (F and R). Sequencing was performed at the LGC Genomics GmbH, Berlin, Germany. The obtained sequences were analysed using the BLASTX search against NCBI’s non-redundant protein database and the *T. bryosalmonae* transcriptome assembly (PRJNA680464, Kumar et al., 2021).

### Gene ontology

The sequences identified by IVIAT were subjected to gene ontology (GO) analysis for biological process, molecular function, and cellular components. GO analysis was performed using Blast2GO version 5.2 software (Gotz et al., 2008). Briefly, the FASTA sequences were subjected to BLASTX in the NCBI non-redundant database with a threshold of E-value 1.0E−3. Subsequently, mapping was done to retrieve the GO terms of the BLAST results. Simultaneously, InterPro annotation was done to retrieve the information regarding protein domain/motif and the obtained information was merged with the already available GO terms. Subsequently, the GO terms were annotated for their functional characteristics.

### Validation of *in vivo*-induced genes

Fourteen genes were selected for validation analysis based on various criteria such as function, involvement in virulence factors and importance in cellular activities. The expression patterns of the genes were validated in infected kidney and infected bryozoan by quantitative real time PCR (qRT-PCR). RNA (n = 6) was extracted from the posterior kidneys of the experimentally infected brown trout (12 wpe) and infected bryozoan *F. sultana* colonies using an RNeasy mini kit (Qiagen) including DNase treatment and was then reverse transcribed to cDNA using iScript cDNA synthesis kit (Bio-Rad). Primers specific for each target gene of *T. bryosalmonae* (**Supplementary Table 1**) were designed using NCBI Primer BLAST tool (http://www.ncbi.nlm.nih.gov/tools/primer-blast/). qRT-PCR was optimized using a temperature gradient. The PCR amplicon of each specific primer was sequenced, and BLAST analysed to ensure their specificity and sensitivity. A CFX96 Touch Real-Time PCR detection system (Bio-Rad) was used to assess the *in vivo* expression of the target genes. qRT-PCR had a final volume of 10 µl, which contained 3 µl of 1:20 fold diluted cDNA, 0.4 μM of each primer, 1X SsoAdvanced™ Universal SYBR Green Supermix (Bio-Rad), and sterile DEPC-treated distilled water. qRT-PCR included initial denaturation at 95°C for 5 minutes, followed by 37 cycles of denaturation at 95°C for 30 seconds, annealing at 60-64°C for 30 seconds, and elongation at 72°C. Final elongation was performed at 95°C for 30 seconds. A melting-point curve analysis was carried from 60–95°C with an increment of 0.5°C per 10 seconds to detect any non-specific PCR products. All samples were analysed in triplicate. The expression level of each tested gene was normalized to the reference genes: 60S ribosomal protein L18 (Gorgoglione et al., 2013) and NADH dehydrogenase. The fold change of *in vivo* induced genes in the infected kidney relative to its infected bryozoan was calculated by the 2^-ΔΔ^ *^Ct^* method (Livak and Schmittgen, 2001). The statistical difference between infected kidney and infected bryozoan samples was determined using the two-tailed unpaired Student’s t-test with Welch’s correction using IBM SPSS software version 25.0.

## Results

### Adsorption of pooled sera

**Figure 2:**
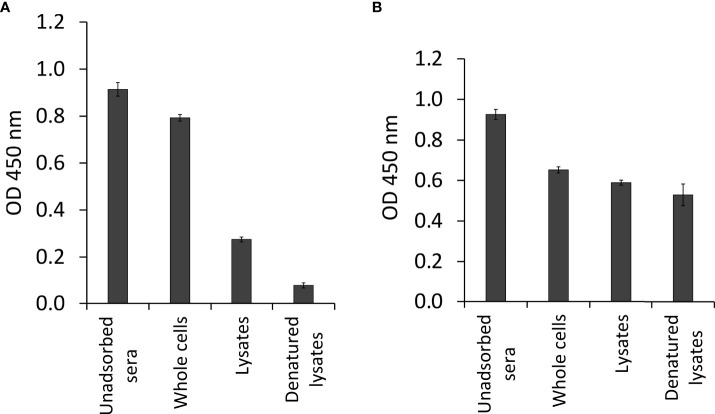
Antibody determination of pooled sera from infected brown trout after sequential adsorption steps by indirect ELISA. **(A)** pooled sera adsorbed by three different forms of *T. bryosalmonae* (native parasite sac whole cells, ultrasonic lysates, and heat-denatured lysates), and **(B)** pooled sera adsorbed by three different forms of *E. coli* XL1-Blue (native bacterial whole cells, ultrasonic lysates, and heat-denatured lysates).

Most of the reductions in OD values of pooled sera were observed after adsorption steps with the parasite sac antigens. OD values from 0.914 to 0.076 were detected for pooled sera diluted 1:100, which was the optimal dilution used for the immunoscreening of the cDNA library. As shown in **Figure 2A**, there was a significant reduction in reactivity of the pooled sera after the parasite sac lysates adsorption step. In contrast, there was considerably less elimination of antibodies recognizing *E. coli* XL1-Blue antigens (**Figure 2B**), suggesting that the adsorption with *E. coli* antigens had no effect on the antibodies against *T. bryosalmonae* in the sera of brown trout.

### *In vivo* induced genes of *T. bryosalmonae*

**Figure 3:**
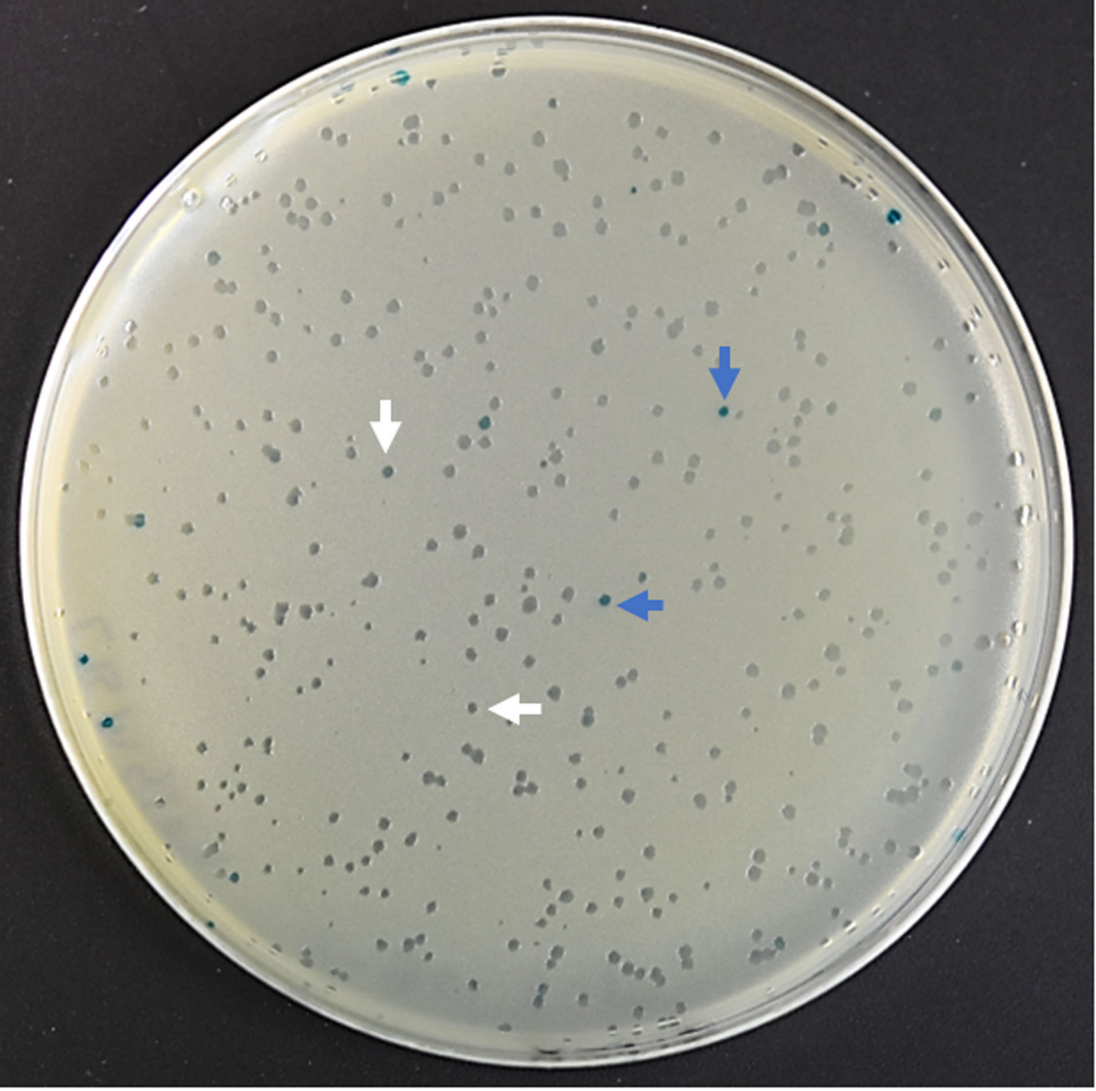
The efficiency of recombinant phage cDNA library. To determine the percentage of recombinant clones, blue–white screening was performed on LB/MgSO4 agar plates containing IPTG and X-gal. White plaque indicates recombinant plaque and blue plaque indicates nonrecombinant plaque.

The phage cDNA library (1.89 x 10^6^ pfu/ml) had a recombination efficiency of 97.88% (**Figure 3**). About 40,000 clones were screened from *T. bryosalmonae* phage cDNA library using the adsorbed pooled positive sera from infected brown trout. A total of 136 clones were identified as immune-reactive; these were then isolated and confirmed by sequencing and NCBI BLAST analysis. The complete list of identified IVI genes by the immunoscreening is presented in **Supplementary Table 2**. Additionally, the functions and locations of some of the identified IVI genes are provided in **Table 1**. PCR products indicated variation in the size of inserts in the cDNA library (**Supplementary Figure 1**).

**Table 1:** Identified antigenic genes of *Tetracapsuloides bryosalmonae*.

*In vivo* induced antigens	Function	Location
Ras-related protein Rab-35	Rab protein signal transduction	Plasma membrane/cytoplasm/nucleous
Calmodulin	Calcium-mediated signaling	Plasma membrane/cytoskeleton
14-3-3-like protein	Signal transduction	Plasma membrane/cytoplasm/nucleous
Transmembrane emp24 domain-containing protein 7	Protein transport	Membrane
Vesicle-associated membrane protein 3-like	Vesicular transport	Plasma membrane
Zinc transporter 1	Zinc transport	Plasma membrane
F-actin-capping protein subunit alpha-2	Actin cytoskeleton organization	Cytosol/extracellular region or secreted/cytoskeleton
Cell division control protein 42 homolog	Actin cytoskeleton organization	Plasma membrane
Endophilin-B1-like	Membrane dynamics	Mitochondrion/Golgi apparatus
Peptidyl-prolyl cis-trans isomerase-like	Protein folding	Cytoplasm
Desumoylating isopeptidase 2	Protein deubiquitination	Cytoplasm
Iron-sulfur cluster assembly enzyme ISCU	Iron-sulfur clusters	Cytoplasm/mitochondrion
Heat shock protein 90	Protein folding	Cytoplasm
COP9 signalosome complex subunit 5	Cellular and developmental processes	Cytoplasm
S-methyl-5’-thioadenosine phosphorylase-like	Purine ribonucleoside salvage	Cytoplasm
Enolase-like	Glycolysis	Cytoplasm
Ceramide synthase 1-like	Sphingolipid biosynthetic pathway	Membrane
Hydroxyacyl-coenzyme A dehydrogenase	Beta-oxidation pathway	Cytoplasm/mitochondrion

*In vivo* induced antigens were identified by immunoscreening of phage cDNA library using pooled adsorbed sera of infected brown trout. Full table is presented in **Supplementary Table 2**.

### Gene ontology

**Figure 4:**
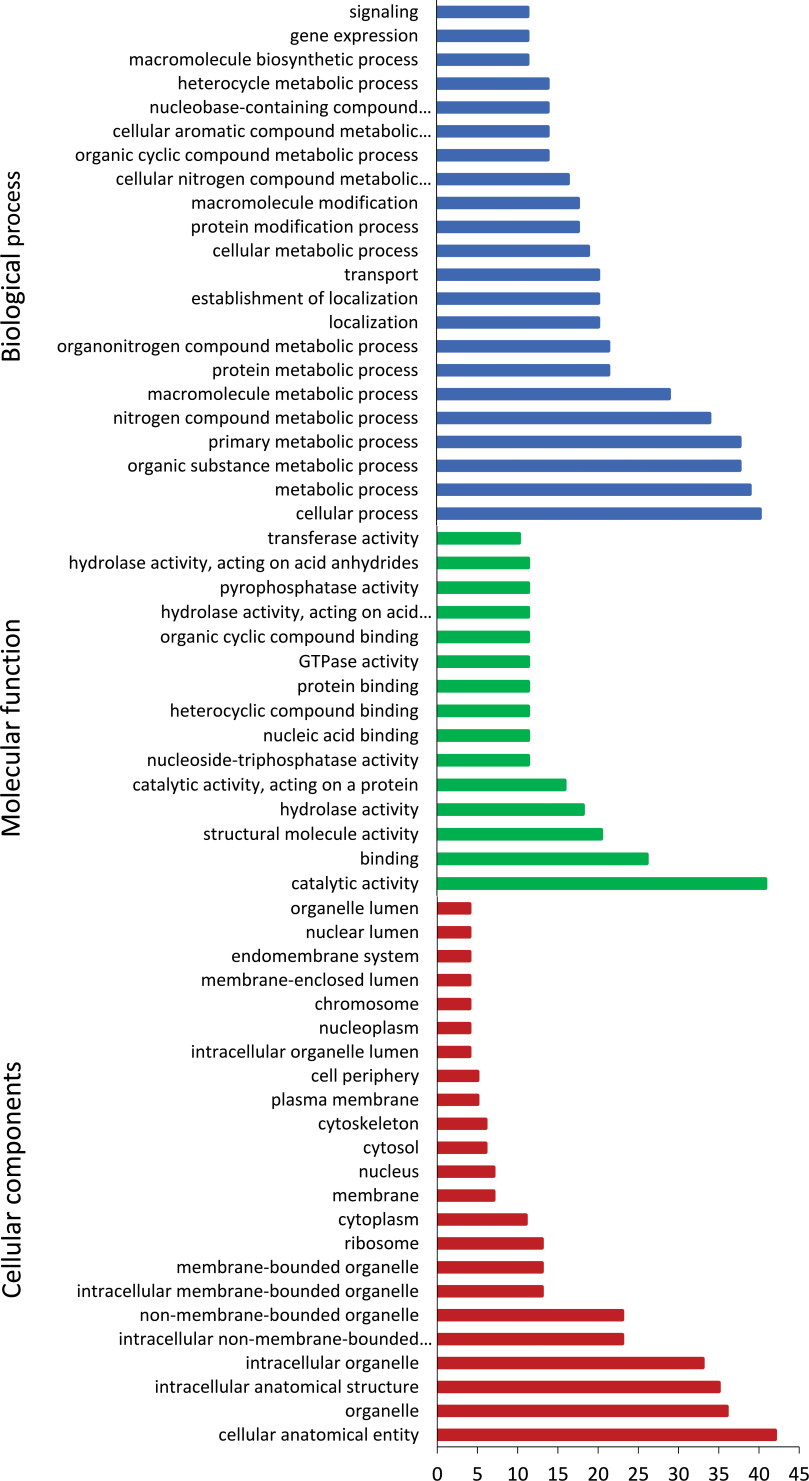
Gene ontology annotation of *Tetracapsuloides bryosalmonae in vivo*-induced genes. Most frequent level 2 GO terms in *T. bryosalmonae* IVI genes, separated for the GO domains; biological process, molecular function, and cellular component.

*In vivo* induced genes were categorised into biological process, molecular function, and cellular components (**Figure 4**). Majority of biological process were associated with the cellular, metabolic, and transport processes. In terms of molecular function, genes were enriched in catabolic activity, binding, and structural molecular activity. Cellular components of genes were abundant in organelle, cytoskeleton, membrane, and ribosome.

### Validation of IVI genes

**Figure 5:**
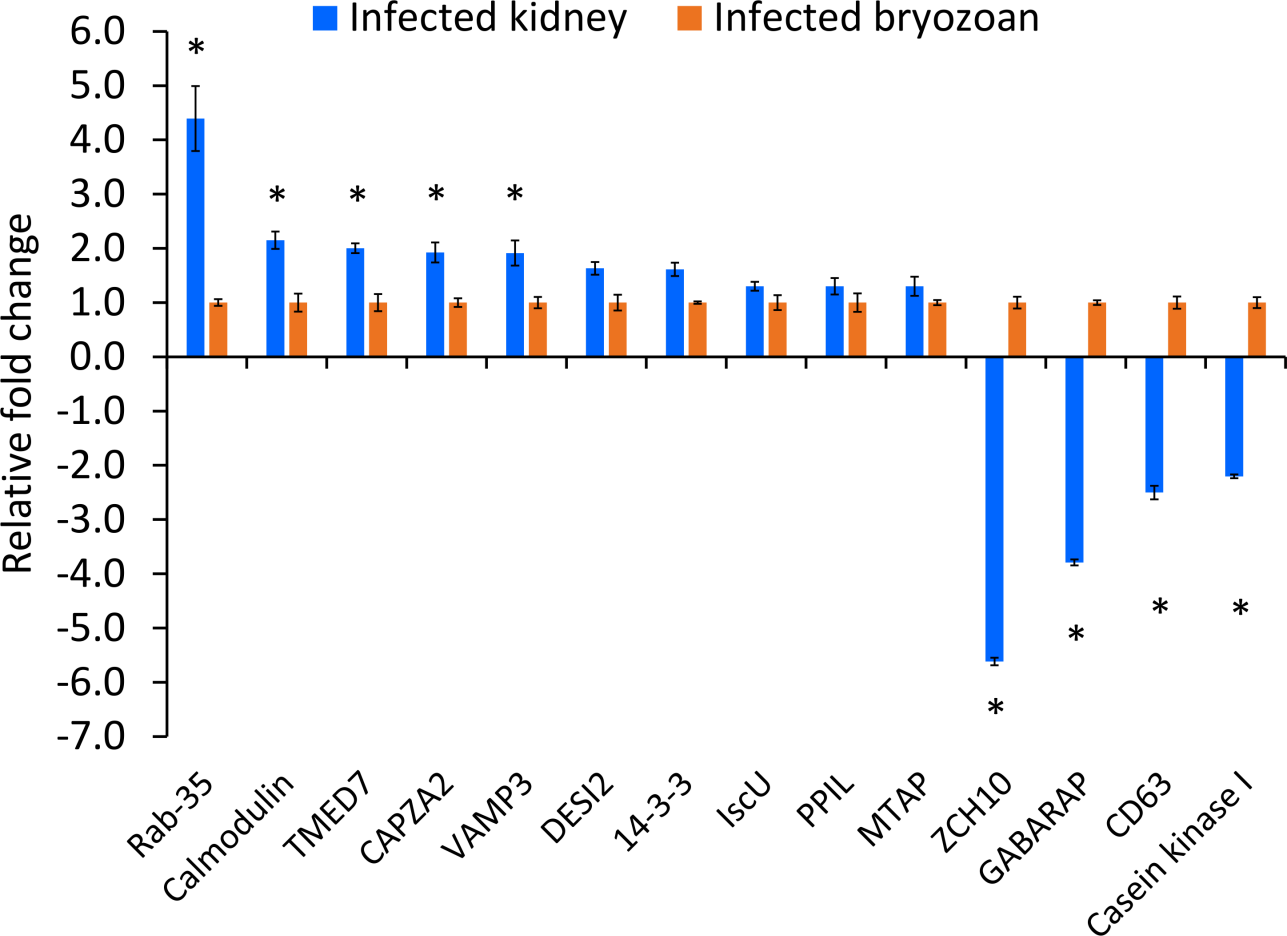
Relative expression of *Tetracapsuloides bryosalmonae in vivo*-induced genes. Assessment of expression of IVI genes in infected kidney of brown trout compared to their expression in infected bryozoan. qRT-PCR data were normalized with reference genes, and represented as a fold change relative to infected bryozoan expression levels. Significant differences (p < 0.05) between infected kidney and infected bryozoan of each gene are indicated with an asterisk (*). Error bars represent the standard error (n = 6). TMED7, Transmembrane emp24 domain-containing protein 7; CAPZA2, F-actin-capping protein subunit alpha-2; VAMP3, Vesicle-associated membrane protein 3; ZCH10, zinc finger CCHC domain-containing protein 10; GABARAP, gamma-aminobutyric acid receptor-associated protein; DESI, desumoylating isopeptidase 2; IscU, iron-sulfur cluster assembly scaffold protein; PPIL, peptidyl-prolyl cis-trans isomerase; MTAP, S-methyl-5’-thioadenosine phosphorylase.

The expression patterns of the fourteen IVI genes in *T. bryosalmonae* dwelling in the infected brown trout kidney and infected bryozoans are shown in **Figure 5**. Five IVI genes were significantly (p < 0.05) upregulated: Ras-related protein Rab-35, calmodulin, transmembrane emp24 domain-containing protein 7, F-actin-capping protein, and vesicle-associated membrane protein 3. Four IVI genes were significantly (p < 0.05) downregulated in *T. bryosalmonae* from infected kidney, compared to their expression in infected bryozoans: zinc finger CCHC domain-containing protein 10, gamma-aminobutyric acid receptor-associated protein, CD63 antigen, and casein kinase I. Additionally, non-significant (p = 0.40) upregulation of five IVI genes (14-3-3-like protein, iron-sulfur cluster assembly scaffold protein, peptidyl-prolyl cis-trans isomerase, S-methyl-5’-thioadenosine phosphorylase, and desumoylating isopeptidase 2) was also observed in the *T. bryosalmonae* from infected kidney compared to infected bryozoans.

## Discussion

The cnidarian parasite *T. bryosalmonae* develops in the kidney of salmonid fishes and can cause devastating disease. The anterior kidney is a major lymphoid organ in fish and has hemopoietic functions. Moreover, both anterior and posterior kidney serve as immunocompetent organs in fish (Kum and Sekki, 2011). In spite of the immune functions of the kidney, *T. bryosalmonae* can successfully undergo sporogony in the kidney of the affected fish and be released in the urine (Sudhagar et al., 2019; Sudhagar et al., 2020) suggesting that this parasite has evolved to survive in an extremely hostile environment by molecular adaption. Most of the previous experiments on brown trout - *T. bryosalmonae* interactions were conducted to understand the host (brown trout) response (Kumar et al., 2014; Kumar et al., 2015; Bailey et al., 2019; Sudhagar et al., 2019; Sudhagar et al., 2020; Shivam et al., 2021; Sudhagar et al., 2022), while only a few studies focused on generating molecular information on *T. bryosalmonae* (Ahmad et al., 2021; Faber et al., 2021; Kumar et al., 2021). Identification of *in vivo* induced antigens or genes of the parasite during host-pathogen interactions, particularly during its development host, will provide valuable insight into the molecular adaption of the parasite. IVIAT has been successfully used to identify antigenic proteins of pathogens expressed during host-pathogen interactions, by using antisera from the infected host (Amerizadeh et al., 2013a; Amerizadeh et al., 2013b; Tao et al., 2014; Ron et al., 2015). In our study, 136 *in vivo* induced genes of *T. bryosalmonae* in brown trout were identified using IVIAT (**Supplementary Table 2**). These genes are involved in a variety of functions critical for the parasite including signal transduction, actin cytoskeleton organization, transport, metal ion binding, transcription, mitochondrial organization, translation, protein folding, metabolic process, cell division, and DNA repair. Additional genes encode for hypothetical proteins, and other genes are uncharacterized. The *in vivo* induced genes identified in this study are antigenic in nature and could potentially be used for the development of vaccines or utilized as drug targets for treating PKD in salmonids. Although there are limitations in the IVIAT technique such as a requirement for cultivable pathogens and an antibody response in the host, it has led to the identification of previously unknown immunogenic antigens of many pathogenic organisms (Rollins et al., 2005).

### Genes involved in signal transduction

We identified several IVI genes involved in signal transduction activity such as ras-related proteins (Rab-35, Rap-1A, Rab-2B, and Ral-B), ras-related and estrogen-regulated growth inhibitor, and ran-specific GTPase-activating protein. Signal transduction is the process by which a cell receives a signal and transmits it to another part of the cell. This can be initiated by either a cell-surface receptor or an intracellular receptor (Bradshaw and Dennis, 2010). During host-parasite interaction; signal transduction mechanisms aid in the molecular cross-talk between the host and the parasite (Lopes-Junior et al., 2022). Rab proteins have an important function in cellular signal transduction and autophagy. Additionally, they are essential for cytokinesis (Kouranti et al., 2006) and act as molecular switches in intracellular membrane trafficking (Li and Marlin, 2015). In the present study, Rab35 was identified as an *in vivo* induced antigen of *T. bryosalmonae*. Gene expression analysis showed *T. bryosalmonae* Rab35 to be upregulated (4.39 fold) in the kidney of infected brown trout. In humans, amoebic colitis is caused by an anaerobic parasitic amoebozoan, *Entamoeba histolytica*. This parasite phagocytoses hosts blood cells and microbiota during pathogenesis. Rab35 is known to regulate erythrophagocytosis of *E. histolytica* in the cup formation and phagosomal maturation (Verma and Datta, 2017). Similarly, developmental stages of *T. bryosalmonae* in the kidney interstitium are commonly found engulfed by host phagocytes (Kent and Hedrick, 1986). The latter researchers found evidence of endocytosis of host material by the parasites migrating to the lumen of the kidney tubules. In addition, during mitotic division of the parasite primary cell, cytokinesis has been demonstrated by ultrastructural analysis (Morris and Adams, 2008). Therefore, we hypothesize a potential role of Rab35 in the cellular processes of endocytosis and cytokinesis in *T. bryosalmonae*.

Another interesting signal transduction molecule we identified was 14-3-3-like protein. Numerous parasites, including *Plasmodium* sp., *Toxoplasma* sp., *Neospora* sp., *Eimeria* sp., *Schistosoma* sp., and *Echinococcus* sp., have been found to contain this highly conserved protein (Siles-Lucas and Gottstein, 2003). In many of these parasites, the protein is suggested to play significant roles during their life cycle. For example, in *Eimeria tenella*, this protein plays an important role in the regulation of mannitol cycle metabolic pathway, which is an important energy source for their sporulation process (Liberator et al., 1998). These proteins modulate protein kinase C (PKC) activity and translocation in *Schistosoma japonicum* and *Schistosoma mansoni*, during their life cycles (Zhang et al., 2000; Siles-Lucas and Gottstein, 2003). Similar to these parasites, 14-3-3 protein might have important roles in sporogony and translocation during the development of *T. bryosalmonae* in its host. Considering its important role, 14-3-3 protein is suggested as a potential vaccine candidate against parasitic infections such as *Schistosoma* sp. and *Echinococcus* sp. (Siles-Lucas and Gottstein, 2003; Siles-Lucas et al., 2008).

### Genes involved in actin cytoskeleton organization

Genes associated with actin cytoskeleton organization that were identified in the present work included actin (cytoplasmic 1 and 2), tubulin (alpha-1 and beta-1), thymosin beta-4, F-actin-capping protein subunit alpha-2 (CAPZA2), and cell division control protein 42 (CDC42). The organization of the actin cytoskeleton is a process performed at the cellular level that results in the assembly, placement, or degradation of the cytoskeleton structure containing actin filaments and other related proteins. Actin is important in a multitude of functions such as the maintenance of shape, structural integrity of the cell, cytokinesis, cellular endocytosis and the transport of membrane vesicles in eukaryotic cells (Pollard and Cooper, 2009; Suarez and Kovar, 2016). In endoparasites like *T. bryosalmonae*, the genes involved in actin cytoskeleton organization might be highly dynamic in their function, particularly when the parasite changes its morphology, shifts within host (one organ to another) and transmits from one host to complete its life cycle. In *M. cerebralis*, a transcript similar to actin related protein 3 homologue (ARP3) was found with a putative function of sporoplasm’s movement regulation during host detection and penetration (Eszterbauer et al., 2009). Furthermore, the genes involved in the cytoskeleton organization (β-actin, Talin and RhoA), and cytoskeletal-extracellular matrix interaction (Integrin-β) were observed to be significantly upregulated in the virulent genotypes of *C. shasta* (Alama-Bermejo et al., 2019; Alama-Bermejo et al., 2020). We also identified an actin protein of *T. bryosalmonae* in the infected kidney of brown trout by anti-parasite antibody-based protein purification followed by mass spectrometry analysis (Kumar et al., 2015). Another study confirmed the important role of actin in cellular motility, which supports the parasite in the evasion from the host immune system (Hartigan et al., 2016). Based on these findings, we speculate that actin cytoskeleton related genes of *T. bryosalmonae* contribute to invasion, host-parasite interaction, growth, migration, and establishment of disease in salmonids.

Small GTPases belonging to the Rho family are important modulators of the actin cytoskeleton. We found Rho family genes of *T. bryosalmonae* such as rho-related GTP-binding protein RhoC, and rho GTPase-activating protein 44-like by immunoscreening analysis. Additionally, CDC42, which belongs to the Rho family of actin regulators, was also revealed in our study. This protein is known to regulate many cell signaling pathways of eukaryotes (Watson et al., 2017). The F-actin-capping protein subunit alpha-2 is another antigenic protein identified by adsorbed sera of infected fish in the present experiment and is known for its role in actin cytoskeleton organization. Capping protein is an alpha/beta heterodimer and it is an important protein in eukaryotes for actin cytoskeleton development (Hart et al., 1997). The role of F-actin has been confirmed in motility and spore formation in *C. shasta* (Brekhman et al., 2021). In light of these reports, and our present study, Rho family genes in *T. bryosalmonae* could play important roles and interact with antibodies of infected fish during the development of the disease. These findings open a new research area on cytoskeleton associated proteins in *T. bryosalmonae*, with the aim of understanding their precise molecular function.

### Genes involved in cellular transportation

Cellular transportation is essential for the normal functioning of a cell to maintain homeostasis. In the current study, we identified zinc transporters such as zinc transporter 1 and zinc transporter 7 as antigenic genes of *T. bryosalmonae*. Zinc acts as a cofactor for various biological systems including cell signaling (Hara et al., 2017), and its deficiency or excess is detrimental to cells (Sugarman, 1983; Wessells et al., 2012). Cells are endowed with zinc transporter proteins to maintain the optimum level of zinc import, intracellular storage and efflux (Dufner-Beattie et al., 2003). In many parasitic organisms, zinc transporters are involved in parasite development and are known virulence factors in *T. gondii* and *L. infantum* (Carvalho et al., 2015; Chasen et al., 2019). In line with these reports, we assume that zinc transporters in *T. bryosalmonae* transport zinc into the cytoplasm and localize to vesicles. However, how zinc transporters contribute in the invasion process of *T. bryosalmonae* needs to be investigated.

Vesicle-associated membrane protein 3-like (VAMP3) of *T. bryosalmonae* was found to be antigenic and upregulated in the infected brown trout (**Figure 5**). VAMP3 is a membrane protein involved in the movement of materials across the cell membrane and it is also necessary for the homeostasis and other physiological processes of the cell (Feldmann et al., 2011). Silencing of VAMP3 affects the migration of cells and cell-mediated adhesion by integrin (Luftman et al., 2009). Furthermore, in the human parasite *Leishmania amazonensis*, VAMP3 has an important function in the formation of parasitophorous vacuoles (Séguin et al., 2022). In the case of malaria parasite, *Plasmodium falciparum* vesicle-mediated trafficking transports parasite proteins into the infected host cytosol and cell membrane (Taraschi et al., 2001). Similarly, transmembrane emp24 domain-containing protein 7 (TMED7) was identified; this protein is involved in cellular transport and is important for the cellular homeostasis (Aber et al., 2019). Currently, there is not much information on membrane proteins of myxozoan parasites, suggesting the need for further studies to understand their molecular and cellular functions.

### Genes involved in protein folding

Heat shock proteins (HSPs) are molecular chaperones that play an essential role in development of parasite and host-parasite interactions (Polla, 1991). HSPs were found to be abundant in the mature spore of *C. shasta* by proteome analysis (Brekhman et al., 2021). HSP60, 70 and 90 were found to be enriched in *T. bryosalmonae* by transcriptome analysis (Faber et al., 2021). By immunoscreening, we found IVI genes associated with HSPs such as HSP12.2, 70, and 90, DNJA1 protein, activator of 90 kDa heat shock protein ATPase homolog 1, and molecular chaperone ABC1 in *T. bryosalmonae*. HSPs have been identified as major antigens of parasitic nematodes and flukes (Arizono et al., 2011; Chung et al., 2017). These findings suggest that HSPs of *T. bryosalmonae* may be involved in the induction of host immunity. We propose that HSPs could be a potential interest for vaccine development or therapeutic target against *T. bryosalmonae*.

While we determined peptidyl-prolyl cis-trans isomerase-like (PPIL) to be antigenic using IVIAT, in qRT-PCR analysis, it was only slightly upregulated (1.30 fold). PPIL molecules are known to have a functional role in host-parasite interaction and anti-parasitic drug action (Bell et al., 2006). Moreover, PPIL plays an important role in the protein folding mechanism, a process making proteins biologically functional. The cis-trans isomerization of peptide bonds N-terminal to proline residues in polypeptide chains is catalysed by peptidyl-prolyl isomerases. This protein folding machinery has been targeted for antagonizing drug resistance in the malaria causing parasite *P. falciparum* (Wilkinson, 2020). Despite the statistically insignificant upregulation of PPIL in our study, in light of its important role and its demonstrated vaccine potential in other parasites, this gene should be further investigated for its functional role and prospective use as a vaccine candidate against *T. bryosalmonae*.

### Genes involved in cellular metabolic processes

Many of the genes identified in this experiment, such as ceramide synthase 1-like (CERS1), COP9 signalosome, hydroxyacyl-coenzyme A dehydrogenase (HADH), and enolase (ENO) have already been shown to have a role in cellular metabolic processes of other parasitic organisms and have been regarded as potential vaccine candidates or drug targets. The CERS1 gene has a crucial role in the sphingolipid biosynthetic pathway and encodes for the catalytic enzyme ceramide synthase that aids in the synthesis of ceramide. In fungal pathogens, ceramide synthase is identified to play an important role in the virulence (Munshi et al., 2018). Ceramide synthase activity is observed in infectious parasites such as *Leishmania* sp. and *T. cruzi* that manipulates host lipidome to favour the establishment of the parasite within the host (Rub et al., 2013). In parasitic (Figueiredo et al., 2012) and fungal pathogens (McEvoy et al., 2020), ceramide synthase is identified as a novel drug target that could interfere with the sphingolipid biosynthetic pathway.

Several parasites such as *Entamoeba histolytica*, *Toxoplasma* sp., *Trypanosoma* sp., and *Leishmania* sp. are known to encode for COP9 signalosome. This gene regulates the ubiquitin-proteasomal system (UPS) protein degradation pathway of *E. histolytica* (Ghosh et al., 2020). Additionally, zinc-ditiocarb complex inhibited *E. histolytica* development by targeting the COP9 signalosome. HADH is an important enzyme involved in the β-oxidation of fatty acids that helps to metabolize lipids as the source of energy. In canines, recombinant HADH protein of *Echinococcus granulosus* offered protection against cystic echinococcosis infection (Xian et al., 2021). Thus, *T. bryosalmonae* might depend on HADH mediated β-oxidation of fatty acids for its energy metabolism. If so, it would be interesting to explore the potential of a recombinant HADH protein of *T. bryosalmonae* as a vaccine candidate to develop prophylactic measures against PKD in salmonids.

The interconversion of 2-phosphoglycerate into phosphoenolpyruvate during glycolysis is mediated by a metalloenzyme ENO (Didiasova et al., 2019). ENO is known to play a key role in enhancing the virulence of trypanosomatid parasites such as *Trypanosoma* sp. and *Leishmania* sp. (Avilán et al., 2011). ENO is highly conserved across species with similar catalytic residues and is identified as a candidate target for antiparasitic drugs against trypanosomatid parasites (Avilán et al., 2011). Hence, experiments are needed to identify potential candidate drugs targeting ENO of *T. bryosalmonae* to treat PKD in salmonids.

Small ubiquitin-like modifier (SUMO) plays an important role in protein modification and regulates a diverse range of cellular processes. Desumoylating isopeptidases are enzymes that reverse protein SUMOylation (Shin et al., 2012). In the present work, desumoylating isopeptidase 2 is identified as an antigenic gene of *T. bryosalmonae* in brown trout. SUMOylation could be potential drug target for treating parasitic diseases (Sumam de Oliveira et al., 2021).

Another interesting protein that we identified was calmodulin, a highly conserved calcium binding protein. Additionally, the result of qRT-PCR confirmed the upregulation of calmodulin in infected kidney during PKD development. Calmodulin regulates various cellular activities such as cell motility, and it has been identified as a part of the invasion motor in *T. gondii* (Nebl et al., 2011). Interestingly, immunogenicity and pathogenicity of calmodulin have been confirmed in *T. gondii* by IVIAT and gene silencing (Tao et al., 2014). In accordance with the above mentioned findings, calmodulin may play an important role as a calcium-binding protein in *T. bryosalmonae* for invasion and motility and could be a possible vaccine candidate against *T. bryosalmonae* infection.

### Genes with unknown function

Among the 136 *in vivo* induced genes of *T. bryosalmonae* identified in the present experiment, we found ten hypothetical proteins. These genes are unique and their functional roles are not known. Further studies are needed to characterize these genes and to understand their role in the pathogenesis of *T. bryosalmonae* in salmonids.

## Conclusion

In this study, we identified *in vivo* induced antigens of *T. bryosalmonae*, a salmonid parasite of economic and ecological significance. This is the first investigation that provides a piece of comprehensive information about the antigenic genes of the parasite during host-pathogen interactions. The identified IVI genes may play critical roles during parasite development and pathogenesis in salmonids. Taken together, the results enhance our understanding of *T. bryosalmonae* virulence mechanism in salmonids. Moreover, this study provides an extensive list of *in vivo* induced genes that could be further evaluated for their potential as drug targets and vaccine candidates against PKD in salmonids.

## Data availability statement

The data presented in the study are deposited in the FigShare repository, accession number 10.6084/m9.figshare.20775592 https://figshare.com/s/dac3158e101e02659af7.

## Ethics statement

The animal study was reviewed and approved by the institutional ethics committee of the University of Veterinary Medicine Vienna and the national authority, according to §26 of the Austrian Law for Animal Experiments, Tierversuchsgesetz 2012 under approval number BMWFW-68.205/0181-WF/V/3b/2017. All methods of this study are reported in accordance to the ARRIVE guidelines for animal research.

## Author contributions

GK conceived and designed the experiments, performed the experiments and wrote the manuscript. AS and SS performed the experiment and reviewed the manuscript. FN, JB, and ME-M designed the experiments and reviewed the manuscript. All the authors approved the final draft.

## Funding

This research was funded by the Austrian Science Fund (FWF) Project No. P 30981-B32 to GK.

## Conflict of interest

The authors declare that the research was conducted in the absence of any commercial or financial relationships that could be construed as a potential conflict of interest.

## Publisher’s note

All claims expressed in this article are solely those of the authors and do not necessarily represent those of their affiliated organizations, or those of the publisher, the editors and the reviewers. Any product that may be evaluated in this article, or claim that may be made by its manufacturer, is not guaranteed or endorsed by the publisher.

## Supplementary material

Supplementary Figure 1 PCR analysis of identified recombinant clones. Gel image showing different sized cDNA inserts from *in vivo* induced positive clones obtained by PCR with vector specific primers. L: 100 bp ladder (Qiagen), Lanes 1–38 PCR-amplified IVI clones and NTC: negative template control.

## References

[B1] Aber R. Chan W. Mugisha S. Jerome-Majewska L. A. (2019). Transmembrane emp24 domain proteins in development and disease. Genet. Res. 101, e14. doi: 10.1017/S0016672319000090 PMC7045115 31878985

[B2] Ahmad F. Debes P. V. Pukk L. Kahar S. Hartikainen H. Gross R. . (2021). Know your enemy - transcriptome of myxozoan *Tetracapsuloides bryosalmonae* reveals potential drug targets against proliferative kidney disease in salmonids. Parasitology 148, 726–739. doi: 10.1017/S003118202100010X 33478602 PMC8056827

[B3] Alama-Bermejo G. Holzer A. S. Bartholomew J. L. (2019). Myxozoan adhesion and virulence: *Ceratonova shasta* on the move. Microorganisms 7, 397. doi: 10.3390/microorganisms7100397 PMC6843538 31561529

[B4] Alama-Bermejo G. Meyer E. Atkinson S. D. Holzer A. S. Wiśniewska M. M. Kolísko M. . (2020). Transcriptome-wide comparisons and virulence gene polymorphisms of host-associated genotypes of the cnidarian parasite *Ceratonova shasta* in salmonids. Genome Biol. Evol. 12, 1258–1276. doi: 10.1093/gbe/evaa109 32467979 PMC7487138

[B5] Amerizadeh A. Idris Z. M. Khoo B. Y. Kotresha D. Yunus M. H. Karim I. Z. . (2013a). Identification of *Toxoplasma gondii in-vivo* induced antigens by cDNA library immunoscreening with chronic toxoplasmosis sera. Microb. Pathog. 54, 60–66. doi: 10.1016/j.micpath.2012.09.006 23044055

[B6] Amerizadeh A. Khoo B. Y. Teh A. Y. Golkar M. Abdul Karim I. Z. Osman S. . (2013b). Identification and real-time expression analysis of selected *Toxoplasma gondii in-vivo* induced antigens recognized by IgG and IgM in sera of acute toxoplasmosis patients. BMC Infect. Dis. 13, 287. doi: 10.1186/1471-2334-13-287 23800344 PMC3695809

[B7] Anderson C. L. Canning E. U. Okamura B. (1999). Molecular data implicate bryozoans as hosts for PKX (Phylum myxozoa) and identify a clade of bryozoan parasites within the myxozoa. Parasitology 119, 555–561. doi: 10.1017/S003118209900520X 10633916

[B8] Arizono N. Yamada M. Tegoshi T. Takaoka Y. Ohta M. Sakaeda T. (2011). Hsp12.6 expression is inducible by host immunity in adult worms of the parasitic nematode *Nippostrongylus brasiliensis* . PloS One 6, e18141. doi: 10.1371/journal.pone.0018141 21448458 PMC3063176

[B9] Avilán L. Gualdrón-López M. Quiñones W. González-González L. Hannaert V. Michels P. A. . (2011). Enolase: A key player in the metabolism and a probable virulence factor of trypanosomatid parasites–perspectives for its use as a therapeutic target. Enzyme Res. 2011, 1–14. doi: 10.4061/2011/932549 PMC3092696 21603223

[B10] Bailey C. Strepparava N. Wahli T. Segner H. (2019). Exploring the immune response, tolerance and resistance in proliferative kidney disease of salmonids. Dev. Comp. Immunol. 90, 165–175. doi: 10.1016/j.dci.2018.09.015 30248359

[B11] Bell A. Monaghan P. Page A. P. (2006). Peptidyl-prolyl cis–trans isomerases (immunophilins) and their roles in parasite biochemistry, host–parasite interaction and antiparasitic drug action. Int. J. Parasitol. 36, 261–276. doi: 10.1016/j.ijpara.2005.11.003 16443228

[B12] Bradshaw R. A. Dennis E. A. (2010). ““Cell signaling,”,” in Handbook of cell signaling, vol. 1–4. (Burlington, MA, USA: Elsevier). doi: 10.1016/B978-0-12-374145-5.00001-2

[B13] Brekhman V. Ofek-Lalzar M. Atkinson S. D. Alama-Bermejo G. Maor-Landaw K. Malik A. . (2021). Proteomic analysis of the parasitic cnidarian *Ceratonova shasta* (Cnidaria: Myxozoa) reveals diverse roles of actin in motility and spore formation. Front. Mar. Sci. 8. doi: 10.3389/fmars.2021.632700

[B14] Carvalho S. Barreira da Silva R. Shawki A. Castro H. Lamy M. Eide D. . (2015). LiZIP3 is a cellular zinc transporter that mediates the tightly regulated import of zinc in *Leishmania infantum* parasites. Mol. Microbiol. 96, 581–595. doi: 10.1111/mmi.12957 25644708 PMC4964273

[B15] Chasen N. M. Stasic A. J. Asady B. Coppens I. Moreno S. (2019). The vacuolar zinc transporter TgZnT protects toxoplasma gondii from zinc toxicity. mSphere 4, e00086–e00019. doi: 10.1128/mSphere.00086-19 31118298 PMC6531880

[B16] Chung E. J. Jeong Y. I. Lee M. R. Kim Y. J. Lee S. E. Cho S. H. . (2017). Heat shock proteins 70 and 90 from *Clonorchis sinensis* induce Th1 response and stimulate antibody production. Parasit. Vectors 10, 118. doi: 10.1186/s13071-017-2026-7 28249599 PMC5333430

[B17] Clifton-Hadley R. Bucke D. Richards R. (1986). Economic importance of proliferative kidney disease of salmonid fish in England and Wales. Vet. Rec. 119, 305–306. doi: 10.1136/vr.119.12.305 3776035

[B18] Dash M. Vasemägi A. (2014). Proliferative kidney disease (PKD) agent *Tetracapsuloides bryosalmonae* in brown trout populations in Estonia. Dis. Aquat. Organ. 109, 139–148. doi: 10.3354/dao02731 24991741

[B19] Didiasova M. Schaefer L. Wygrecka M. (2019). When place matters: shuttling of enolase-1 across cellular compartments. Front. Cell Dev. Biol. 7. doi: 10.3389/fcell.2019.00061 PMC6498095 31106201

[B20] Dörfler C. El-Matbouli M. (2007). Isolation of a subtilisin-like serine protease gene (MyxSubtSP) from spores of *Myxobolus cerebralis*, the causative agent of whirling disease. Dis. Aquat. Organ. 73, 245–251. doi: 10.3354/dao073245 17330744

[B21] Dufner-Beattie J. Langmade S. J. Wang F. Eide D. Andrews G. K. (2003). Structure, function, and regulation of a subfamily of mouse zinc transporter genes. J. Biol. Chem. 278, 50142–50150. doi: 10.1074/jbc.M304163200 14525987

[B22] Eszterbauer E. Kallert D. M. Grabner D. El-Matbouli M. (2009). Differentially expressed parasite genes involved in host recognition and invasion of the triactinomyxon stage of *Myxobolus cerebralis* (Myxozoa). Parasitology 136, 367–377. doi: 10.1017/S0031182008005398 19195410

[B23] Faber M. Shaw S. Yoon S. de Paiva Alves E. Wang B. Qi Z. . (2021). Comparative transcriptomics and host-specific parasite gene expression profiles inform on drivers of proliferative kidney disease. Sci. Rep. 11, 2149. doi: 10.1038/s41598-020-77881-7 33495500 PMC7835236

[B24] Feist S. W. Longshaw M. Canning E. U. Okamura B. (2001). Induction of proliferative kidney disease (PKD) in rainbow trout *Oncorhynchus mykiss via* the bryozoan *Fredericella sultana* infected with *Tetracapsula bryosalmonae* . Dis. Aquat. Organ. 45, 61–68. doi: 10.3354/dao045061 11411645

[B25] Feldmann A. Amphornrat J. Schonherr M. Winterstein C. Mobius W. Ruhwedel T. . (2011). Transport of the major myelin proteolipid protein is directed by VAMP3 and VAMP7. J. Neurosci. 31, 5659–5672. doi: 10.1523/JNEUROSCI.6638-10.2011 21490207 PMC6622839

[B26] Figueiredo J. M. Rodrigues D. C. Silva R. C. M. C. Koeller C. M. Jiang J. C. Jazwinski S. M. . (2012). Molecular and functional characterization of the ceramide synthase from trypanosoma cruzi. Mol. Biochem. Parasitol. 182, 62–74. doi: 10.1016/j.molbiopara.2011.12.006 22226824 PMC3551351

[B27] Ghosh S. Farr L. Singh A. Leaton L. A. Padalia J. Shirley D. A. . (2020). COP9 signalosome is an essential and druggable parasite target that regulates protein degradation. PloS Pathog. 16, e1008952. doi: 10.1371/journal.ppat.1008952 32960936 PMC7531848

[B28] Gorgoglione B. Wang T. Secombes C. J. Holland J. W. (2013). Immune gene expression profiling of proliferative kidney disease in rainbow trout *Oncorhynchus mykiss* reveals a dominance of anti-inflammatory, antibody and Th cell-like activities. Vet. Res. 44, 55. doi: 10.1016/j.fsi.2013.03.061 23865616 PMC3733943

[B29] Gotz S. Garcia-Gomez J. M. Terol J. Williams T. D. Nagaraj S. H. Nueda M. J. . (2008). High-throughput functional annotation and data mining with the Blast2GO suite. Nucleic Acids Res. 36, 3420–3435. doi: 10.1093/nar/gkn176 18445632 PMC2425479

[B30] Hara T. Takeda T. A. Takagishi T. Fukue K. Kambe T. Fukada T. (2017). Physiological roles of zinc transporters: molecular and genetic importance in zinc homeostasis. J. Physiol. Sci. 67, 283–301. doi: 10.1007/s12576-017-0521-4 28130681 PMC10717645

[B31] Hartigan A. Estensoro I. Vancová M. Bílý T. Patra S. Eszterbauer E. (2016). New cell motility model observed in parasitic cnidarian *Sphaerospora molnari* (Myxozoa: Myxosporea) blood stages in fish. Sci. Rep. 6, 39093. doi: 10.1038/srep39093 27982057 PMC5159882

[B32] Hart M. C. Korshunova Y. O. Cooper J. A. (1997). Vertebrates have conserved capping protein α isoforms with specific expression patterns. Cell Motil. Cytoskeleton 38, 120–132. doi: 10.1002/(SICI)1097-0169(1997)38:2<120::AID-CM2>3.0.CO;2-B 9331217

[B33] Hedrick R. P. MacConnell E. de Kinkelin P. (1993). Proliferative kidney disease of salmonid fish. Annu. Rev. Fish Dis. 3, 277–290. doi: 10.1016/0959-8030(93)90039-E

[B34] Hutchins P. R. Sepulveda A. J. Hartikainen H. Staigmiller K. D. Opitz S. T. Yamamoto R. M. . (2021). Exploration of the 2016 Yellowstone river fish kill and proliferative kidney disease in wild fish populations. Ecosphere 12, e03436. doi: 10.1002/ecs2.3436

[B35] Kelley G. O. Adkison M. A. Leutenegger C. M. Hedrick R. P. (2003). *Myxobolus cerebralis*: identification of a cathepsin z-like protease gene (MyxCP-1) expressed during parasite development in rainbow trout, *Oncorhynchus mykiss* . Exp. Parasitol. 105, 201–210. doi: 10.1016/j.exppara.2003.12.004 14990313

[B36] Kelley G. Zagmutt-Vergara F. Leutenegger C. Adkison M. Baxa D. Hedrick R. (2004). Identification of a serine protease gene expressed by *Myxobolus cerebralis* during development in rainbow trout *Oncorhynchus mykiss* . Dis. Aquat. Organ. 59, 235–248. doi: 10.3354/dao059235 15264720

[B37] Kent M. L. Hedrick R. P. (1986). Development of the PKX myxosporean in rainbow trout salmo gairdneri. Dis. Aquat. Organ. 1, 169–182. doi: 10.3354/dao001169

[B38] Kouranti I. Sachse M. Arouche N. Goud B. Echard A. (2006). Rab35 regulates an endocytic recycling pathway essential for the terminal steps of cytokinesis. Curr. Biol. 16, 1719–1725. doi: 10.1016/j.cub.2006.07.020 16950109

[B39] Kumar G. Abd-Elfattah A. El-Matbouli M. (2014). Differential modulation of host genes in the kidney of brown trout *Salmo trutta* during sporogenesis of *Tetracapsuloides bryosalmonae* (Myxozoa). Vet. Res. 45, 101. doi: 10.1186/s13567-014-0101-z 25297457 PMC4198790

[B40] Kumar G. Abd-Elfattah A. Saleh M. El-Matbouli M. (2013). Fate of *Tetracapsuloides bryosalmonae* (Myxozoa) after infection of brown trout *Salmo trutta* and rainbow trout *Oncorhynchus mykiss* . Dis. Aquat. Organ. 107, 9–18. doi: 10.3354/dao02665 24270019 PMC3962845

[B41] Kumar G. Ertl R. Nilsen F. Bartholomew J. L. El-Matbouli M. (2021). Data of *de novo* transcriptome assembly of the myxozoan parasite *Tetracapsuloides bryosalmonae* . Data Brief 35, 106831. doi: 10.1016/j.dib.2021.106831 33659593 PMC7890133

[B42] Kumar G. Gotesman M. El-Matbouli M. (2015). Interaction of *Tetracapsuloides bryosalmonae*, the causative agent of proliferative kidney disease, with host proteins in the kidney of *Salmo trutta* . Parasitol. Res. 114, 1721–1727. doi: 10.1007/s00436-015-4357-7 25663070 PMC4412511

[B43] Kum C. Sekki S. (2011). “The immune system drugs in fish: Immune function, immunoassay, drugs,” in Recent advances in fish farms. Eds. Aral F. Doğu Z. (London: IntechOpen Press), 169–216.

[B44] Liberator P. Anderson J. Feiglin M. Sardana M. Griffin P. Schmatz D. . (1998). Molecular cloning and functional expression of mannitol-1-phosphatase from the apicomplexan parasite *Eimeria tenella* . J. Biol. Chem. 273, 4237–4244. doi: 10.1074/jbc.273.7.4237 9461622

[B45] Li G. Marlin M. C. (2015). Rab family of GTPases. Mol. Biol. 1298, 1–15. doi: 10.1007/978-1-4939-2569-8_1 PMC5903570 25800828

[B46] Livak K. J. Schmittgen T. D. (2001). Analysis of relative gene expression data using real-time quantitative PCR and the 2(-delta delta C(T)) method. Methods 25, 402–408. doi: 10.1006/meth.2001.1262 11846609

[B47] Lopes-Junior E. H. Marques R. P. Bertevello C. R. Oliveira K. C. (2022). “Perspective chapter: Molecular crosstalk and signal transduction between platyhelminths and their hosts,” in Parasitic helminths and zoonoses - from basic to applied research. Eds. Morales-Montor J. Araiza V. H. D. R. (London: IntechOpen Press).

[B48] Luftman K. Hasan N. Day P. Hardee D. Hu C. (2009). Silencing of VAMP3 inhibits cell migration and integrin-mediated adhesion. Biochem. Biophys. Res. Commun. 380, 65–70. doi: 10.1016/j.bbrc.2009.01.036 19159614 PMC2716655

[B49] McEvoy K. Normile T. G. Poeta M. D. (2020). Antifungal drug development: targeting the fungal sphingolipid pathway. J. Fungi 6, 142. doi: 10.3390/jof6030142 PMC7559796 32825250

[B50] Morris D. J. Adams A. (2008). Sporogony of *Tetracapsuloides bryosalmonae* in the brown trout *Salmo trutta* and the role of the tertiary cell during the vertebrate phase of myxozoan life cycles. Parasitology 135, 1075–1092. doi: 10.1017/S0031182008004605 18549518

[B51] Munshi M. A. Gardin J. M. Singh A. Luberto C. Rieger R. Bouklas T. . (2018). The role of ceramide synthases in the pathogenicity of *Cryptococcus neoformans* . Cell Rep. 22, 1392–1400. doi: 10.1016/j.celrep.2018.01.035 29425496 PMC5839121

[B52] Nebl T. Prieto J. H. Kapp E. Smith B. J. Williams M. J. Yates J. R. 3rd . (2011). Quantitative *in vivo* analyses reveal calcium-dependent phosphorylation sites and identifies a novel component of the *Toxoplasma* invasion motor complex. PloS Pathog. 7, e1002222. doi: 10.1371/journal.ppat.1002222 21980283 PMC3182922

[B53] Okamura B. Hartikainen H. Schmidt-Posthaus H. Wahli T. (2011). Life cycle complexity, environmental change and the emerging status of salmonid proliferative kidney disease. Freshw. Biol. 56, 735–753. doi: 10.1111/j.1365-2427.2010.02465.x

[B54] Polla B. S. (1991). Heat shock proteins in host-parasite interactions. Immunol. Today 12, A38–A41. doi: 10.1016/S0167-5699(05)80011-8 2069677

[B55] Pollard T. D. Cooper J. A. (2009). Actin, a central player in cell shape and movement. Science 326, 1208–1212. doi: 10.1126/science.1175862 19965462 PMC3677050

[B56] Rollins S. M. Peppercorn A. Hang L. Hillman J. D. Calderwood S. B. Handfield M. . (2005). *In vivo* induced antigen technology (IVIAT). Cell. Microbiol. 7, 1–9. doi: 10.1111/j.1462-5822.2004.00477.x 15617518

[B57] Ron M. Gorelick-Ashkenazi A. Levisohn S. Nir-Paz R. Geary S. J. Tulman E. . (2015). *Mycoplasma gallisepticum in vivo* induced antigens expressed during infection in chickens. Vet. Microbiol. 175, 265–274. doi: 10.1016/j.vetmic.2014.12.007 25575879

[B58] Rub A. Arish M. Husain S. A. Ahmed N. Akhter Y. (2013). Host-lipidome as a potential target of protozoan parasites. Microbes Infect. 15, 649–660. doi: 10.1016/j.micinf.2013.06.006 23811020

[B59] Séguin O. Mai L. T. Acevedo Ospina H. Guay-Vincent M. M. Whiteheart S. W. Stäger S. . (2022). VAMP3 and VAMP8 regulate the development and functionality of parasitophorous vacuoles housing *Leishmania amazonensis* . Infect. Immun. 90, e0018321. doi: 10.1128/iai.00183-21 35130453 PMC8929380

[B60] Shin E. J. Shin H. M. Nam E. Kim W. S. Kim J. Oh B. H. . (2012). DeSUMOylating isopeptidase: a second class of SUMO protease. EMBO Rep. 13, 339–346. doi: 10.1038/embor.2012.3 22370726 PMC3321169

[B61] Shivam S. El-Matbouli M. Kumar G. (2021). Kinetics of parasite-specific antibody and b-cell-associated gene expression in brown trout, *Salmo trutta* during proliferative kidney disease. Biology 10, 1244. doi: 10.3390/biology10121244 34943159 PMC8698486

[B62] Siles-Lucas M. Gottstein B. (2003). The 14-3-3 protein: a key molecule in parasites as in other organisms. Trends Parasitol. 19, 575–581. doi: 10.1016/j.pt.2003.10.003 14642768

[B63] Siles-Lucas M. Merli M. Gottstein B. (2008). 14-3-3 proteins in *Echinococcus*: Their role and potential as protective antigens. Exp. Parasitol. 119, 516–523. doi: 10.1016/j.exppara.2008.01.009 18316081

[B64] Skovgaard A. Buchmann K. (2012). *Tetracapsuloides bryosalmonae* and PKD in juvenile wild salmonids in Denmark. Dis. Aquat. Organ. 101, 33–42. doi: 10.3354/dao02502 23047189

[B65] Sterud E. Forseth T. Ugedal O. Poppe T. T. Jørgensen A. Bruheim T. . (2007). Severe mortality in wild Atlantic salmon *Salmo salar* due to proliferative kidney disease (PKD) caused by *Tetracapsuloides bryosalmonae* (Myxozoa). Dis. Aquat. Organ. 77, 191–198. doi: 10.3354/dao01846 18062470

[B66] Suarez C. Kovar D. R. (2016). Internetwork competition for monomers governs actin cytoskeleton organization. Nat. Rev. Mol. Cell Biol. 17, 799–810. doi: 10.1038/nrm.2016.106 27625321 PMC5125073

[B67] Sudhagar A. El-Matbouli M. Kumar G. (2020). Identification and expression profiling of toll-like receptors of brown trout (*Salmo trutta*) during proliferative kidney disease. Int. J. Mol. Sci. 21, 3755. doi: 10.3390/ijms21113755 PMC7312180 32466538

[B68] Sudhagar A. El-Matbouli M. Kumar G. (2022). Genome-wide alternative splicing profile in the posterior kidney of brown trout (*Salmo trutta*) during proliferative kidney disease. BMC Genomics 23, 446. doi: 10.1186/s12864-022-08685-4 35710345 PMC9204890

[B69] Sudhagar A. Ertl R. Kumar G. El-Matbouli M. (2019). Transcriptome profiling of posterior kidney of brown trout, *Salmo trutta*, during proliferative kidney disease. Parasit. Vectors 12, 569. doi: 10.1186/s13071-019-3823-y 31783772 PMC6884850

[B70] Sugarman B. (1983). Zinc and infection. Rev. Infect. Dis. 5, 137–147. doi: 10.1093/clinids/5.1.137 6338570

[B71] Sumam de Oliveira D. Kronenberger T. Palmisano G. Wrenger C. de Souza E. E. (2021). Targeting SUMOylation in plasmodium as a potential target for malaria therapy. Front. Cell. Infect. Microbiol. 11. doi: 10.3389/fcimb.2021.685866 PMC8224225 34178724

[B72] Tao Q. Xiao J. Wang Y. Fang K. Li N. Hu M. . (2014). Identification of genes expressed during toxoplasma gondii infection by *in vivo*-induced antigen technology (IVIAT) with positive porcine sera. J. Parasitol. 100, 470–479. doi: 10.1645/13-240.1 24646180

[B73] Taraschi T. F. Trelka D. Martinez S. Schneider T. O’Donnell M. E. (2001). Vesicle-mediated trafficking of parasite proteins to the host cell cytosol and erythrocyte surface membrane in *Plasmodium falciparum* infected erythrocytes. Int. J. Parasitol. 31, 1381–1391. doi: 10.1016/S0020-7519(01)00256-9 11566305

[B74] Vasemägi A. Nousiainen I. Saura A. Vähä J. P. Valjus J. Huusko A. (2017). First record of proliferative kidney disease agent *Tetracapsuloides bryosalmonae* in wild brown trout and European grayling in Finland. Dis. Aquat. Org. 125, 73–78. doi: 10.3354/dao03126 28627494

[B75] Verma K. Datta S. (2017). The monomeric GTPase Rab35 regulates phagocytic cup formation and phagosomal maturation in *Entamoeba histolytica* . J. Biol. Chem. 292, 4960–4975. doi: 10.1074/jbc.M117.775007 28126902 PMC5377809

[B76] Wahli T. Knuesel R. Bernet D. Segner H. Pugovkin D. Burkhardt-Holm P. . (2002). Proliferative kidney disease in Switzerland: Current state of knowledge. J. Fish Dis. 25, 491–500. doi: 10.1046/j.1365-2761.2002.00401.x

[B77] Waldner K. Bechter T. Auer S. Borgwardt F. El-Matbouli M. Unfer G. (2020). A brown trout (*Salmo trutta*) population faces devastating consequences due to proliferative kidney disease and temperature increase: A case study from Austria. Ecol. Freshw. Fish 29, 465–476. doi: 10.1111/eff.12528

[B78] Watson J. R. Owen D. Mott H. R. (2017). Cdc42 in actin dynamics: An ordered pathway governed by complex equilibria and directional effector handover. Small GTPases 8, 237–244. doi: 10.1080/21541248.2016.1215657 27715449 PMC5680673

[B79] Wessells K. R. Singh G. M. Brown K. H. (2012). Estimating the global prevalence of inadequate zinc intake from national food balance sheets: Effects of methodological assumptions. PloS One 7, e50565. doi: 10.1371/journal.pone.0050565 23209781 PMC3510064

[B80] Wilkinson M. D. (2020). Targeting protein folding in the malaria parasite (London, UK: Imperial College London).

[B81] Xian J. Wang N. Zhao P. Zhang Y. Meng J. Ma X. . (2021). Molecular characterization and immune protection of the 3-hydroxyacyl-CoA dehydrogenase gene in *Echinococcus granulosus* . Parasit. Vectors 14, 489. doi: 10.1186/s13071-021-05001-z 34556147 PMC8460197

[B82] Zhang Y. Bickle Q. D. Taylor M. G. (2000). Cloning of *Schistosoma japonicum* 14-3-3 epsilon (Sj14-3-3 epsilon), a new member of the 14-3-3 family of proteins from schistosomes. Int. J. Parasitol. 30, 991–994. doi: 10.1016/s0020-7519(00)00086-2 10980288

