# Supplementary material for: Identification of in vivo induced antigens of the malacosporean parasite Tetracapsuloides bryosalmonae (Cnidaria) using in vivo induced antigen technology
Source: Front Cell Infect Microbiol. 2022 Oct 26;12:1032347. doi: 10.3389/fcimb.2022.1032347 (PMC9644027; doi:10.3389/fcimb.2022.1032347)
Supplement: Supplementary file 1 [file Table_1.docx]

**Supplementary Table 1**: Nucleotide sequence of quantitative real-time PCR primers used in this study.

| Gene name | Code | Sequence | Annealing temp (°C) | Amplicon size (bp) |
| --- | --- | --- | --- | --- |
| Ras-related protein Rab-35-like | Rab-35 F2 | AGCCTACTTCTTCGATTTTCTGAG | 62 | 185 |
|  | Rab-35 R2 | ACACCATGCGTTCCTCGATA |  |  |
| Calmodulin | Cal F | TAGCATCAGCATCAACTTCGTTA | 60 | 151 |
|  | Cal R | GCAGAGTTTAAGGAAGCTTTCAGT |  |  |
| Transmembrane emp24 domain-containing protein 7 | TMED7 F2 | CGCCTCGATAAATGGACCTAAC | 60 | 153 |
|  | TMED7 R2 | GAGACTTCACGAAAGCAAACTGA |  |  |
| F-actin-capping protein subunit alpha-2 | CAPZA F2 | TTACGAAGATGGAAACGTTCAGT | 60 | 197 |
|  | CAPZA R2 | CTGGTAGTTGACGTCGTAGC |  |  |
| Vesicle-associated membrane protein 3-like | VAMP3 F2 | GCTGATGCCCTACAACGTG | 60 | 160 |
|  | VAMP3 R2 | GAGTTACACTGACTGAGACCCA |  |  |
| 14-3-3-like protein | 14-3-3 F2 | ATAGCAAGACAGAGAGAGCCG | 62 | 179 |
|  | 14-3-3 R2 | AGCCGTCTTGGCTAATTCAC |  |  |
| Iron-sulfur cluster assembly scaffold protein IscU | IscU F | TGTGGTTCAGCTATTGCATCTTC | 60 | 132 |
|  | IscU R | GCAATGCAGTTTAACAGGTGGT |  |  |
| Peptidyl-prolyl cis-trans isomerase-like | PPIL F | AACAGCAGAAAATTTCAGGGCT | 60 | 135 |
|  | PPIL R | TTTCCTCCTGTTCCGTTGTGA |  |  |
| S-methyl-5'-thioadenosine phosphorylase-like | MTAP F | AGCTGAAAGCAAAATGTTTCAAAGT | 60 | 125 |
|  | MTAP R | CATAGTCTGTGACGAGGCCA |  |  |
| Desumoylating isopeptidase 2 | DI 2 F | GGACTGACGGATGTGTTACTG | 60 | 164 |
|  | DI 2 R | GTCCGTAGACTTCCAATCCACT |  |  |
| zinc finger CCHC domain-containing protein 10-like | ZCH10 F | GACGTAAGCTCTTGGATGTGAT | 60 | 175 |
|  | ZCH10 R | TCTTAGTGGAAGAAGGTCTGTGA |  |  |
| gamma-aminobutyric acid receptor-associated protein | GABARAP F2 | ACCAGTGATTGTTGAGAAAGTTCC | 60 | 191 |
|  | GABARAP R2 | GTCCCATAGTCATGGAGGTAGA |  |  |
| CD63 antigen | CD63 F2 | TGGTGAAATTGAATACGGAGGTC | 62 | 198 |
|  | CD63 R2 | ATGGCTGAGCCCACTAAAGA |  |  |
| Casein kinase I | Casein kinase I F | TGGAAAATCTTGTAACAAAGTCCAC | 60 | 166 |
|  | Casein kinase I R | ACGTCTTGACTGTTCAATTCCAA |  |  |
| NADH dehydrogenase [ubiquinone] iron-sulfur protein 2 | NADH 2 F | CCGTGCCACGATGTAAAAGT | 60 | 197 |
|  | NADH 2 R | AGAATTTTTGGGTGCTGCCG |  |  |
| 60S ribosomal protein L18 * | RPL18 F | GTAAACGGGGACAAAAAGA | 60 | 251 |
|  | RPL18 R | GGAGCAGCACCAAAATAC |  |  |

* Gorgoglione, B., Wang, T., Secombes, C. J., and Holland, J. W. (2013). Immune gene expression profiling of proliferative kidney disease in rainbow trout *Oncorhynchus mykiss* reveals a dominance of anti-inflammatory, antibody and Th cell-like activities. *Vet. Res.* 44, 55. doi: 10.1016/j.fsi.2013.03.061.
