# Supplementary figures and images for: Identification of in vivo induced antigens of the malacosporean parasite Tetracapsuloides bryosalmonae (Cnidaria) using in vivo induced antigen technology
Source: Front Cell Infect Microbiol. 2022 Oct 26;12:1032347. doi: 10.3389/fcimb.2022.1032347 (PMC9644027; doi:10.3389/fcimb.2022.1032347)

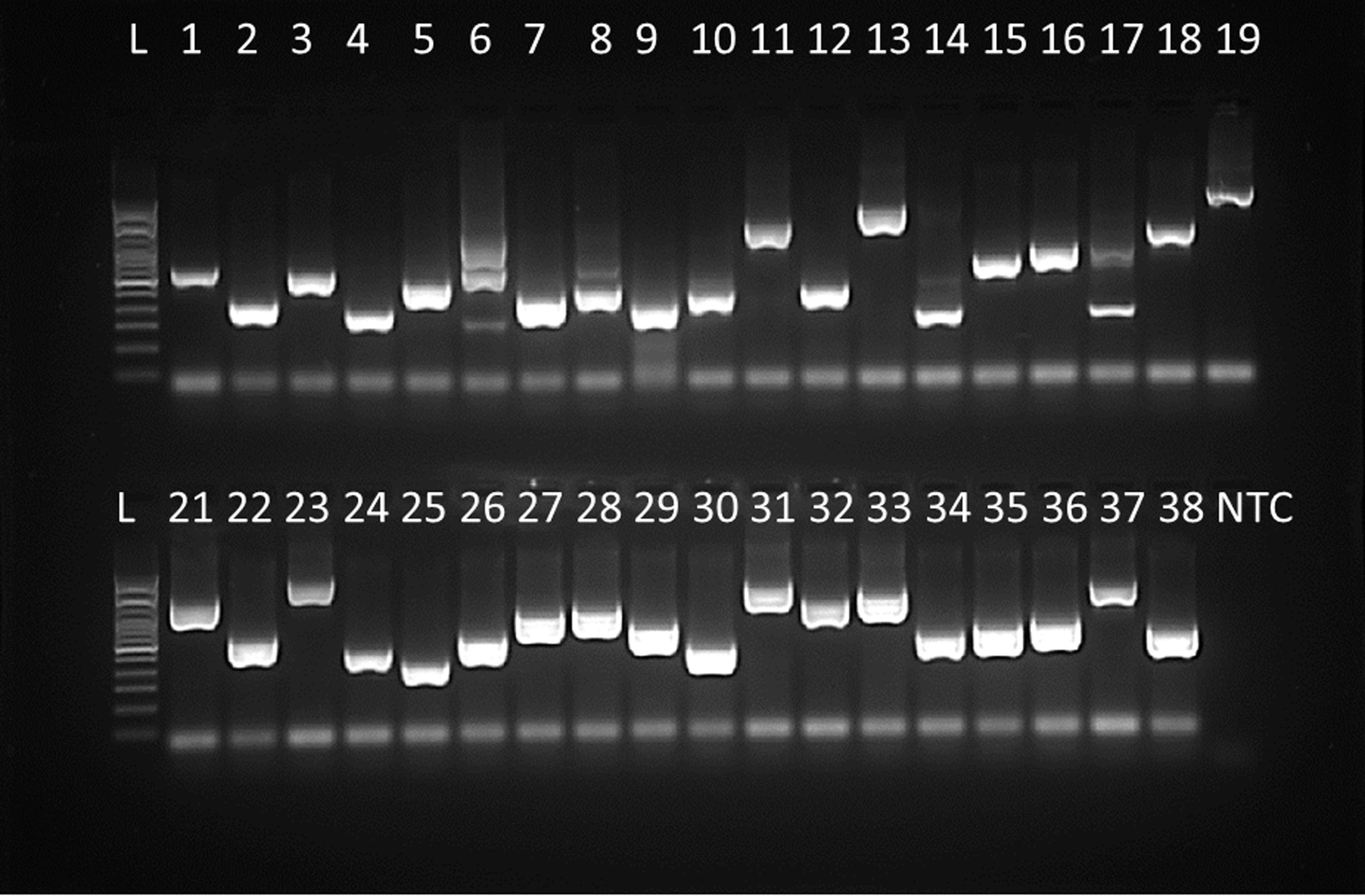

Supplement: Supplementary Figure 1 — PCR analysis of identified recombinant clones. Gel image showing different sized cDNA inserts from in vivo induced positive clones obtained by PCR with vector specific primers. L: 100 bp ladder (Qiagen), Lanes 1–38 PCR-amplified IVI clones and NTC: negative template control. [file Image_1.tif]
